# Abstract of the Proceedings of the Buffalo Medical Association

**Published:** 1856-04

**Authors:** Sanford B. Hunt


### BUFFALO MEDICAL JOURNAL

AND

### MONTHLY REVIEW.

VOL. 11. APRIL, 1856. NO. 11.

### ORIGINAL COMMUNICATIONS.

ART. I.—*Abstract of the Proceedings of the Buffalo Medical Association.*

The meeting adjourned to the evening of Wednesday, February 20th, to consider the subject of a new organization, was not held, owing to failure to attend.

The regular meeting for March was held on the evening of Tuesday, the 4th instant.

Present—Drs. Strong, Baker, Wilcox, Eastman, Mixer, Hamilton, Hunt, Wyckoff, Gray, Newman, White, Flint, Howell, Underhill, and Gould.

The minutes of the preceding meeting were read and approved.

Drs. C. L. Dayton, B. H. Lemon, George Hadley, and S. O. Almy, were then unanimously elected to membership in the association.

*Dr. Hamilton* presented to the society a specimen of Felt Splint which he had had manufactured in Buffalo, and which was equal to any he had before seen. It is quite plastic when exposed to heat, and becomes very firm when cool. It is much cheaper than gutta percha, and it is not liable to be injured by keeping; it wears out, also, less rapidly, when in use, than gutta percha.

Dr. H. presented an apparatus, made of leather, for the treatment of fractures of the inferior maxilla—the same which is described and figured in his report on fractures, in the Transactions of the National Association for 1855. Also, a modification of Fox's apparatus for broken clavicle; a simple straight splint for broken thighs in children, and a more complex splint, with a worm screw for making extension. Dr. H. wished, however, to call especial attention to his long, straight splint, in which he has employed Dr. Neill's principle of extension and counter-extension, but so modified as to render its operation more exact and effectual. By this apparatus extension and counter-extension are effected at the same moment, by the action of a screw at the lower extremity of the splint.

Dr. Hamilton received the suggestion of the principle from the very ingenius apparatus devised by Dr. John Neill, Prof. of Surgery in Pennsylvania Med. Col., Philadelphia; but he finds the principle has been applied in a rude way from a very early period, and in Scultetus Surgery it is called Galen's *Glossocomon:* of which Galen says it was "invented by the latter Physitians." It was used, however, only to enable the assistants to transfer the patient from one bed to another. *Vide* "*The Chirurgeons Store-House,*" *by Johannes Scultetus, of Ulme, in Suaovia. London.* 1674. p. 52. The description of the apparatus is, in the volume referred to, accompanied with a drawing representing the apparatus applied and worked with a wench. Dr. Neill uses a Spanish windlass, and Dr. Hamilton a screw. It would be difficult to explain the mode of its operation without a drawing, but to those who have seen Dr. Neill's apparatus, it will be sufficient to say that counter-extension is made by moving the *foot* piece toward the lower end of the splint—the counter-extending band being fastened to the *outer* end of the foot piece, as it is projected from within outward through the splint, while the extension is effected at the same moment through the attachment of the foot to the *inner* end of the same foot piece.

Mr. King, druggist, will hereafter keep these various splints for sale, or will order them of his manufacturer, Mr. Arnold. It is unnecessary to say, that no one will disturb any patent by making for himself or for others, any of these kinds of apparatus, and that Dr. Hamilton has no interest in any way with their manufacture or sale.

On inquiry of the President as to prevalent diseases, pneumonia and pneumonic or bronchial difficulties were mentioned as prevalent by all the members; erysipelas, by Drs. Wilcox, Strong and Wyckoff; scarlatina, by Drs. White, Gray, Wyckoff and others; small-pox, by Dr. White; and typhoid fever by several members. The type of the various contagious or exanthematous diseases mentioned, was stated to be mild, with very few exceptions, and none of them were stated to be prevailing epidemically.

Among the individual cases of interest reported, Dr. Gray mentioned two cases of mild scarlatina in children, which followed eight or ten days after an adult in the family had had a sore throat resembling that of scarlatina, presenting, in fact, all the characteristics of the disease as presented in the fauces, but without any eruption upon the skin. A question was raised as to the connection, by contagion, of the cases of the children with that of the adult.

*Dr. Wyckoff* mentioned that four individuals, in one family, had been attacked by severe headache, which continued two or three days. At the end of that time, a vesicular eruption appeared upon the scalp, which dried up, became scaly, and then presented an eczematous appearance. In one of the cases erysipelas of the face succeeded the vesicular eruption. The family attributed the disease to the use of bay-rum.

*Dr. Strong* related the case of a man who called at his office, suffering from intense headache, with the prodromic symptoms of fever. The patient had had pleurisy during the season, and had also suffered from some form of renal difficulty.

On the day succeeding, the patient took to his bed with violent febrile action, presenting during two or three days some evidences of periodicity. As the symptoms did not yield to antiperiodic treatment, he was then inclined to regard it as continued fever, and did so for three or four days longer. At the end of this time, the patient was taken with profuse discharges from the bowels of a choleraic character, accompanied by vomitings of a serous fluid. The evacuations were similar to those of cholera, frequent and profuse, but were controlled during twenty-four hours by the usual remedies. At this time he was removed to the hospital for better care, but, soon after his admission, had a return of the diarrhœa and vomiting, of the same character as that previously described, he sank with collapse, his voice was husky and whispering as in cholera, he had urgent thirst, and died on the third day after his removal. He mentioned the case as being remarkably choleraic in its character.

*Prof. Flint* inquired whether there had been any œdema of the face or extremities. (Answered in the negative.) Dr. F. said, that his motive in inquiring was to ascertain whether there had been any evidences of Bright's disease, the fact of some renal difficulty having been mentioned. He had once seen a case presenting similar symptoms, in which Bright's disease was found upon a *post-mortem* examination, though it had not been suspected before.

Dr. Flint also mentioned, while speaking, that he had witnessed, during his service at the Louisville Marine Hospital, the past winter, a well-marked case of complete relapse of typhoid fever. The patient passed through the ordinary phenomena of typhoid in about the usual time, but, after a convalescence of a fortnight's duration, he again took to his bed, and passed through the disease again, presenting the eruption and other symptoms in their usual order. The patient recovered.

*Dr. Mixer* reported a case of foreign body in the œsophagus. The patient, a gentleman at a htoel, presented the symptoms of suffocative cough, with profuse expectoration of blood, and very great distress. On passing down the finger he found a fish bone in the œsophagus, below the line of the lower border of the larynx, firmly imbedded across the passage. He passed down a pair of curved forceps and seized it, but could not grasp it firmly enough to remove it, the shape of the bone being such that the forceps slipped. He then passed down a director beside his finger, and, pushing away the wall of the œsophagus from the bone, he was enabled to raise one end and place it diagonally across the tube. The other end was then detached in the same manner, and brought up, and by a repetition of this movement it was loosened so that it was expelled by a paroxysm of coughing. The whole operation occupied nearly half an hour. The bone was a flat one, representing two sides of a square, each $\frac{3}{4}$ of an inch long, and sharp pointed.

*Prof. Hamilton* made some practical remarks on the subject of foreign bodies in the œsophagus. It was, perhaps, his fault, or his misfortune, to be frequently reporting unfortunate cases. This habit, if injurious to himself, was, at any rate, beneficial to others, and he would not forego it on the present occasion. He wished to warn those not familiar with them, against the use of blunt hooks in extracting foreign bodies from the œsophagus; and would illustrate by two cases. In one, he passed the flat hook beyond the body, but was then unable either to bring up the body, or to detach the hook. After half an hour's labor, with great suffering to the patient, he succeeded in removing the hook by the use of great force, inflicting, undoubtedly, serious injury on the patient.

In his second case, he used the "button instrument," and caught it beyond the foreign body, and had much difficulty in removing it. The patient fell from the chair upon the floor, and for the moment he thought her dead. He thought that in the majority of cases the foreign body could not be got out. "Bond's forceps" were also objectionable, and he thought the finger the only instrument that could by safely used.

*Dr. Wyckoff* gave a brief history of a case in which he removed a fish bone, $\frac{3}{4}$ of an inch wide by $1\frac{1}{4}$ inch long, from the anus, where it was imbedded crosswise — one end was sharp.

*Dr. Baker* mentioned a case in which a girl inhaled a brass tack into the *left* bronchus. It remained there for three years, and was finally expelled in a paroxysm of cough. She had been supposed to have consumption, but recovered her health after the expulsion of the tack. This case is given in Gross' work on Foreign Bodies in the Air Passages.

Dr. Baker proceeded to report, from the committee on organization, a series of by-laws for the contemplated new organization.

*Dr. Hunt* inquired whether the "Buffalo Medical Association" was competent to adopt by-laws for the "Buffalo Medical and Surgical Association."

*Dr. Newman*, on the part of the committee, related the history of the formation of the committee, stated the laws governing similar organizations, and discussed briefly the necessity of a legal organization, as a corporation entitled to hold property.

He concluded by moving that when we adjourn we do so to Tuesday next. *Carried.*

*Prof. Hamilton* moved, That Dr. John Boardman be appointed a committee to report on the "Lodgement of Foreign Bodies in the Alimentary Passages."

Dr. Hamilton remarked that he had some reason to suppose that the various hair dyes, now so much in use, were producing injurious effects upon the hair, permanently staining the skin, and even occasionally affecting injuriously the health of the persons using them. He moved, therefore, that Dr. George Hadley, Prof. of Chemistry, etc., be appointed a committee to report on the subject of Hair Dyes, and to determine whether any of them are capable of producing these effects. *Carried.*

And the association then adjourned.

SANFORD B. HUNT, Secretary.

